# Preliminary Structural Characterization of an Anti-Inflammatory Polysaccharide from *Lysimachia christinae* and Its Preventive Effect on Lipopolysaccharide-Induced Acute Liver Injury in Mice

**DOI:** 10.3390/molecules31193432

**Published:** 2026-09-27

**Authors:** Yuanyuan Zhang, Zhou Hong, Xiwei Jia, Meimei Zhang, Hao Yang, Liming Zhang, Lishuang Zhou

**Affiliations:** 1 School of Pharmacy, Ningxia Medical University, Yinchuan 750004, China; zyynxmu@163.com (Y.Z.); yhao246@163.com (H.Y.); 2 College of Life Science and Technology, Ningxia Vocational and Technical University, Yinchuan 750021, China; hongzhou0503@163.com (Z.H.); gslzjxw@163.com (X.J.); 3 School of Basic Medical Science, Ningxia Medical University, Yinchuan 750004, China; zhangmm196@163.com; 4 School of Ocean and Tropical Medicine, Guangdong Medical University, Zhanjiang 524023, China

**Keywords:** *Lysimachia christinae* Hance, polysaccharide, structural characterization, anti-inflammatory, acute liver injury

## Abstract

An acidic heteropolysaccharide, named LCP-90-3, with a relative molecular weight of 14.02 kDa, was isolated and purified from the traditional heat-clearing Chinese medicine, *Lysimachia christinae* Hance. The monosaccharide residue analysis indicated that LCP-90-3 was composed of glucuronic acid, mannose, and arabinose in a molar ratio of 79.2: 3.6: 17.2. Linkage analysis further indicated that LCP-90-3 was mainly composed of 1,4-Glc*p*A, 1,4,6-Glc*p*A, 1,2-Ara*f*, T-Man*p*, 1,3-Ara*f*, and 1,4-Man*p* residues. Furthermore, LCP-90-3 demonstrated potent anti-inflammatory activity by significantly inhibiting the secretion of nitric oxide (NO), tumor necrosis factor-α (TNF-α), and interleukin-6 (IL-6) in lipopolysaccharide (LPS)-treated ANA-1 cells. Interestingly, oral administration of LCP-90-3 at doses of 20, 40, and 80 mg/kg effectively mitigated LPS-induced acute liver injury *in vivo* by suppressing the overexpression of inflammatory mediators (NO, TNF-α, and IL-6), aminotransferases (ALT and AST) in serum, and markers of antioxidant capacity (MDA, CAT, and SOD) in the liver. Overall, these findings highlight LCP-90-3 as a novel leading polysaccharide for new drug candidate development against liver injury through modulation of inflammatory responses and oxidative stress.

## 1. Introduction

The liver is a vital metabolic and detoxifying organ in the human body. Currently, liver injury has become a major global health concern, characterized by high mortality rates and poor prognosis [1]. The etiology of liver injury is multifactorial, encompassing viral infections, drug toxicity, alcohol abuse, and chemical exposure [2]. Pathologically, liver injury involves a cascade of complex processes, including inflammatory responses, oxidative stress, inflammatory cell infiltration, apoptotic cell death, and dysregulated autophagy [3,4]. Notably, excessive release of inflammatory mediators has been identified as a central pathogenic mechanism underlying most acute liver injury cases [5]. Given the current lack of definitive protective agents in clinical practice, the exploration and development of natural hepatoprotective products with enhanced safety profiles, minimal toxicity, and reduced adverse effects has become an urgent research priority in hepatology.

Traditional Chinese Medicine (TCM) exhibits unique protective advantages in hepatic disorders through its multi-targeted mechanisms of action. These mechanisms achieve comprehensive protective outcomes via coordinated metabolic regulation, inflammation modulation, and functional restoration of hepatic tissue [6]. Among the diverse bioactive components in TCM, polysaccharides have been pharmacologically validated as potent hepatoprotective agents with multidimensional activities [7]. For instance, *Schisandra chinensis*-derived acidic polysaccharides ameliorate ethanol-induced hepatotoxicity by suppressing CYP2E1-dependent oxidative pathways [8]. Additionally, *Plantago asiatica* polysaccharides exhibit dual detoxification mechanisms against acetaminophen toxicity through hepatic–gut metabolic crosstalk modulation and intestinal microbiome reconstitution [9]. Collectively, these findings highlight the potential of TCM poly-saccharides in developing safe and effective protectives for acute liver injury. Our preliminary investigations have extended this paradigm to heat-clearing herbs. Polysaccharides isolated from *Houttuynia cordata*, *Tamarix chinensis*, and *Hedyotis diffusa* exhibited significant mitigation of virus- or lipopolysaccharide (LPS)-induced organ damage in murine models, primarily through anti-inflammatory pathways and immune homeostasis regulation [10,11,12]. This evidence positions polysaccharides from heat-clearing TCM herbs as particularly promising candidates for addressing LPS-induced acute liver injury, warranting further systematic exploration.

*Lysimachia christinae* Hance, a classical heat-clearing herb in TCM, has long been used in decoction form for treating hepatic inflammation and urolithiasis [13]. While its flavonoid constituents, phenolic acids, and crude extracts have demonstrated well-characterized antioxidant and anti-inflammatory properties [7,14], the hepatoprotective potential of its polysaccharides remains unexplored. In our preliminary studies, we successfully isolated an acidic heteropolysaccharide (designated LCP-90-1) from this herb, which exhibited significant *in vitro* anti-complementary activity and free-radical-scavenging capacity [15]. These findings prompted us to systematically investigate whether *L. christinae* polysaccharides possess anti-inflammatory efficacy and protective potential against hepatitis.

In this study, we report the bioactivity-guided isolation and purification of LCP-90-3 from *L. christinae* using DEAE-cellulose chromatography. This acidic heteropolysaccharide was further evaluated for its anti-inflammatory and hepatoprotective properties. Our findings provide insights into the potential molecular basis underlying the preventive effects of LCP-90-3, and offer a scientific basis for developing novel anti-inflammatory or hepatoprotective agents against acute liver injury.

## 2. Results

### 2.1. Isolation, Purification, Homogeneity, and Relative Molecular Weight of LCP-90-3

The crude polysaccharide LCP-90 (50 g) was obtained from 15 kg dried *L. christinae* via water extraction and ethanol precipitation. LCP-90 was fractionated on a DEAE-52 column to yield the fraction eluted with 0.4 M NaCl, designated LCP-90-3 (629.14 mg), as shown in Figure 1. The HPGPC-ELSD (Figure 2A) and HPGPC-RID (Figure 2B) chromatograms of LCP-90-3 each displayed a single, symmetrical peak. The calibration curves for weight-average molecular weight (*Mw*) and number-average molecular weight (*Mn*) were y = −0.5033x + 11.872 (R^2^ = 0.9982) and y = −0.5204x + 12.123 (R^2^ = 0.9951), respectively, where y is Log *Mw* or Log *Mn* and x is the retention time. Using a retention time of 15.345 min for LCP-90-3, the corresponding *Mw* and *Mn* were calculated to be 1.40 × 10^4^ Da and 1.37 × 10^4^ Da, respectively. The polydispersity index (PDI = *Mw*/*Mn*) was 1.02, indicating that LCP-90-3 has a narrow molecular weight distribution (Figure S2). In addition, the contents of uronic acid, total carbohydrate, and protein were 77.82 ± 0.09%, 93.16 ± 0.89%, and 1.80 ± 0.04%, respectively.

### 2.2. Structural Characterization of LCP-90-3

Monosaccharide composition analysis showed that LCP-90-3 was mainly composed of glucuronic acid (79.2%), mannose (3.6%), and arabinose (17.2%) in a molar ratio of 79.2: 3.6: 17.2 (Figure 2C,D).

The FT-TR spectrum of LCP-90-3 (Figure 3A) suggested that the broad band at 3331 cm^−1^ corresponded to the O-H stretching vibration [12]. The signal at 2919 cm^−1^ was attributed to C-H stretching vibration [16]. Additionally, the absorbance peaks at 1555 cm^−1^ and 1388 cm^−1^ were associated with the asymmetrical and symmetrical stretching vibrations of COO^−^ in the carboxyl groups, indicating the presence of uronic acids [17]. Several absorptions near 1000–1200 cm^−1^ were linked to C-O stretching frequencies [18]. These results suggested that LCP-90-3 exhibited typical FT-IR absorption characteristic of polysaccharides.

To determine the glycosyl linkage types, uronic acid in LCP-90-3 was reduced prior to methylation, resulting in the carboxyl-reduced polysaccharide designated as LCP-90-3R. FT-IR analysis of the methylated LCP-90-3R showed that the hydroxyl band near 3400 cm^−1^ almost disappeared after methylation (Figure 3B), confirming the complete methylation of LCP-90-3R. Subsequently, the methylated products of LCP-90-3R were hydrolyzed, reduced, and acetylated to yield their permethylated alditol acetates (PMAAs). The results of GC-MS analysis of PMAAs are presented in Figure 4A,B, with characteristic fragmentation patterns listed in Table 1. The PMAAs of LCP-90-3R comprised six components, which include 1,2-Ara*f*, T-Man*p*, 1,3-Ara*f*, 1,4-Man*p*, 1,4-Glc*p*, and 1,4,6-Glc*p* in molar ratios of 21.4: 0.9: 1.1: 2.2: 68.7: 5.7. Due to the reduction of GlcA, the connection modes of LCP-90-3 included 1,2-Ara*f*, T-Man*p*, 1,3-Ara*f*, 1,4-Man*p*, 1,4-Glc*p*A, and 1,4,6-Glc*p*A, with the same molar ratio of 21.4: 0.9: 1.1: 2.2: 68.7: 5.7.

### 2.3. Anti-Inflammatory Activity of LCP-90-3 in LPS-Stimulated ANA-1 In Vitro

To examine the effects of LCP-90-3 on ANA-1 cell viability, the cells were exposed to different concentrations (500, 250, 125, 62.5, and 31.25 μg/mL) of LCP-90-3 for 24 h, and cell viability was measured by MTT assay [19]. As shown in Figure 5A, LCP-90-3 exhibited no significant toxicity toward ANA-1 cells.

The effect of LCP-90-3 on the expression of NO, TNF-α, and IL-6 in LPS-stimulated ANA-1 cells was evaluated using ELISA. As shown in Figure 5B–D, the levels of NO, TNF-α, and IL-6 were all significantly increased in the LPS model group compared with the control group (*p* < 0.001). However, the levels of NO, TNF-α, and IL-6 were significantly reduced in the LCP-09-3 (125, 250, and 500 μg/mL)- and DEX (60 μg/mL)-treated groups (*p* < 0.01). Moreover, with the increase in the dose of LCP-90-3, the inhibitory effect on the inflammatory mediators of LPS-stimulated ANA-1 cells was stronger.

### 2.4. Protective Effect of LCP-90-3 Against LPS-Induced Acute Liver Injury in Mice

#### 2.4.1. LCP-90-3 Inhibited the Expression of Inflammatory Mediators in Mice

To assess the effect of LCP-90-3 on inflammatory response in mice with LPS-induced acute liver injury, ELISA was performed to detect the levels of NO, TNF-α, and IL-6 in serum. As shown in Figure 6A–C, the levels of NO, TNF-α, and IL-6 were all significantly increased in the LPS model group compared with the control group (*p* < 0.001). In contrast, the levels of NO, TNF-α, and IL-6 in mice treated with LCP-09-3 (40 and 80 mg/kg) and silymarin (80 mg/kg) were all significantly reduced (*p* < 0.01). These results indicated that LCP-90-3 could exert anti-inflammatory effects *in vivo* by suppressing the overproduction of systemic inflammatory cytokines in mice with LPS-induced acute liver injury.

#### 2.4.2. LCP-90-3 Improved the Antioxidant Capacity in Liver

To assess the effect of LCP-90-3 on liver antioxidant capacity in mice with LPS-induced acute liver injury, the MDA level and the activities of CAT and SOD in liver were detected. As displayed in Figure 6D–F, compared with the control group, the activities of CAT and SOD were all significantly decreased in the LPS model group (*p* < 0.001), while the liver MDA level was increased (*p* < 0.001). Compared with the LPS group, the LCP-09-3 (40 and 80 mg/kg) and silymarin (80 mg/kg) groups all showed significantly increased activities of CAT and SOD, and a remarkable reduction in MDA level.

#### 2.4.3. LCP-90-3 Decreased the Levels of Indicators for Liver Function in Mice

To assess the effect of LCP-90-3 on liver function in mice with LPS-induced acute liver injury, the activities of ALT and AST in serum were detected. As illustrated in Figure 6G,H, the activities of ALT and AST in serum were all significantly increased in the LPS model group compared with the control group (*p* < 0.01). Compared with the LPS model group, the LCP-09-3 (40 and 80 mg/kg) and silymarin (80 mg/kg) groups exhibited significantly decreased activities of ALT and AST (*p* < 0.05). 

#### 2.4.4. LCP-90-3 Alleviated the Degree of Liver Damage in Mice

The result of histologic examination in Figure 7. H&E staining revealed that LPS led to bleeding, necrosis, and inflammatory cell infiltration in hepatic lobules. Concurrently, LCP-90-3 and silymarin exerted a certain degree of protective effect on mice with liver injury.

## 3. Discussion

The occurrence of liver injury is a complex process driven by multiple etiological factors (e.g., viral infections, chemical intoxication) and core pathological mechanisms, including dysregulated inflammatory responses, oxidative stress, and immune imbalance [20,21]. Notably, uncontrolled inflammation acts as a central hub in acute liver injury progression: pathogens or toxins activate innate immune cells, triggering signaling pathways that induce the excessive release of pro-inflammatory cytokines (TNF-α and IL-6) and reactive oxygen species (ROS). These mediators further induce hepatocyte necrosis, apoptosis, and impaired tissue repair [20,21]. Currently, there is a lack of clinically available protective agents specifically targeting the pathological mechanisms underlying liver injury. Given the dominant role of inflammatory cascades in acute liver injury, developing natural anti-inflammatory agents with favorable safety profiles has become a critical research focus. Recent studies demonstrate that polysaccharides from heat-clearing traditional Chinese medicines (TCMs) can mitigate viral- and LPS-induced organ damage in murine models through anti-inflammatory and immunomodulatory pathways [22,23,24]. These findings highlight the preventive potential of polysaccharides isolated from heat-clearing herbs for the treatment of LPS-induced acute liver injury.

As a classical heat-clearing herb, *Lysimachia christinae* has been traditionally used as an aqueous decoction for treating hepatic inflammation and urinary calculi. Considering that polysaccharides are essential bioactive components in Chinese herbs and their water-solubility aligns with traditional decoction methods, we hypothesize that polysaccharides might be the main active ingredients responsible for the hepatoprotective effects of *L. christinae*. In this study, we isolated and purified an acidic polysaccharide from *L. chritinae*, designated as LCP-90-3. Linkage analysis showed that LCP-90-3 mainly contained 1,4-GlcpA residues, which accounted for 68.7% of total glycosidic linkages, and systematically evaluated its anti-inflammatory and hepatoprotective activities.

ANA-1 macrophage experiments revealed that LCP-90-3 exhibited no cytotoxicity and significantly suppressed LPS-induced overexpression of inflammatory mediators (NO, TNF-α, and IL-6) in a dose-dependent manner. Notably, its inhibitory efficacy was comparable to that of the positive control dexamethasone (DEX), confirming its potent *in vitro* anti-inflammatory activity. In the experiment of LPS-induced acute liver injury in mice, LCP-90-3 could significantly reduce the expression levels of NO, TNF-α, and IL-6 in serum, indicating that LCP-90-3 exerts anti-inflammatory activity *in vivo* by inhibiting the overexpression of these inflammatory mediators, thereby contributing to its protective effect. Further analyses revealed that LCP-90-3 significantly attenuated LPS-induced elevations in serum hepatocellular damage biomarkers (ALT and AST) [25] and modulated hepatic antioxidant indices (MDA, CAT, and SOD) [26]. Specifically, LCP-90-3 reduced oxidative stress by lowering MDA levels and enhancing CAT/SOD activities. Collectively, these results suggest that LCP-90-3 ameliorates LPS-induced acute liver injury, accompanied by reduced inflammatory cytokine release and alleviated oxidative stress. 

Although LCP-90-3 showed slightly weaker hepatoprotective and antioxidant effects compared to silymarin, its superior anti-inflammatory activity suggests distinct protective advantages. This unique profile positions LCP-90-3 as a promising candidate for developing anti-inflammatory agents targeting liver injury, particularly in inflammation-dominant pathologies. In addition, *Oudemansiella radicata* polysaccharides (ORP), with glucose as the main composition, exhibits antioxidant, anti-apoptotic, and TLR4/NF-κB-mediated anti-inflammatory effects in the LPS-induced liver injury model. However, its inhibitory potency against TNF-α/IL-6 is weaker than that of the positive control [27]. In contrast, LCP-90-3, a novel glucuronic acid-type polysaccharide first reported in this study, shows anti-inflammatory efficacy comparable to the positive drug at high doses, indicating a potent ability to block the inflammatory cascade. On the other hand, *Ilex asprella* polysaccharide (IAP), another glucose-type polysaccharide, reduces endotoxin entry into the bloodstream through a synergistic “anti-inflammation—intestinal barrier” mechanism [28]. This alleviates hepatic inflammatory burden at the source, forming a favorable complement to the signaling pathway-mediated action of LCP-90-3. Additionally, crude polysaccharides from *Rehmannia glutinosa* [29], *Arctium lappa* [30], and other sources have also been confirmed to exert hepatoprotective activities through relevant mechanisms. Notably, LCP-90-3, reported in this study, is a novel polysaccharide for the prevention and treatment of liver injury, mainly composed of glucuronic acid, and its characteristics can provide guidance for the screening of compounds of the same type.

This study still has certain limitations, and future in-depth explorations will focus on the following directions: (1) Chemical structure analysis: A complete and clear structural diagram of the target polysaccharide has not yet been explicitly provided. In the future, we will continuously optimize the structural analysis strategy, and accurately determine its glycosidic bond linkage patterns, branching sites, and spatial conformation by combining multiple complementary techniques such as nuclear magnetic resonance (NMR). Ultimately, a clear and accurate chemical structure diagram will be provided to lay a structural foundation for subsequent research. (2) Elucidation of mechanism of action: This study has confirmed that the polysaccharide exerts a clear protective effect against acute liver injury, but the mechanism research still needs to be deepened. Firstly, the specific molecular pathways through which it regulates liver injury repair have not been fully clarified. Secondly, the similarities and differences in the mechanism of action between this polysaccharide and positive control drugs (such as silymarin) have not been systematically compared and analyzed. We will gradually clarify the above scientific issues through proteomics, metabolomics, molecular docking and other technologies, and improve the mechanism network of its hepatoprotective effect. Moreover, endotoxin contamination of LCP-90-3 was not directly measured in this study. Since LPS-stimulated macrophages were used in the *in vitro* assays, this should be considered an important limitation. Future studies will include endotoxin testing of LCP-90-3 to further validate its anti-inflammatory activity. Finally, it should be noted that histopathological evaluation in this study was descriptive and based on representative H&E-stained sections. No blinded semi-quantitative scoring or quantitative morphometric analysis was performed. Therefore, the histological findings should be interpreted as qualitative observations rather than quantitative evidence of histological improvement. (3) Exploration of structure-activity relationship: Two homogeneous polysaccharides (LCP-90-1 and LCP-90-3) isolated from this plant both exhibit strong anti-inflammatory activity, and they share a common structural feature—their glucuronic acid (GlcA) content both exceeds 50%. This suggests that high GlcA content may be the key structural domain for its activity, but direct evidence is currently lacking. Meanwhile, apart from GlcA, the contribution and synergistic mechanism of other monosaccharide components (such as arabinose, mannose, etc.) to hepatoprotective activity remain unclear. In the future, we will construct a correlation model of “structural characteristics-activity intensity” through experiments such as chemical modification (e.g., partial degradation of GlcA residues), structural transformation, and activity verification. This will clarify the functional value of each monosaccharide component and provide a theoretical basis for the structural optimization of polysaccharide-based hepatoprotective drugs.

## 4. Materials and Methods

### 4.1. Materials and Reagents

The whole herb of *Lysimachia christinae* Hance was purchased from Bozhou Medicinal Materials Market (Bozhou, China). Cellulose DEAE-52 was purchased from Whatman Co., Ltd. (Maidstone, Kent, UK). Dextran standards (P5, P10, P20, P50, P100, P200, P400, and P800) were purchased from Shodex (Yokohama, Japan). Monosaccharide standards including Man, Rha, GlcA, GalA, Glc, Gal, Xyl, and Ara were obtained from Sigma-Aldrich (St. Louis, MO, USA). Dexamethasone sodium phosphate (DEX) was obtained from Jiangxi Chuang Xin Pharmaceutical Group Co., Ltd. (Zhangshu, China). Silymarin (purity > 80%) was obtained from Aladdin (Shanghai, China). And other chemicals of analytical grade were purchased from the Sinopharm Chemical Reagent Co., Ltd. (Shanghai, China) and Sigma-Aldrich (St. Louis, MO, USA).

Macrophage ANA-1 cells of mouse were obtained from Procell Life Science & Technology (Wuhan, China). RPMI 1640 medium and fetal bovine serum (FBS) were purchased from GIBCO (New York, NY, USA). Lipopolysaccharide (LPS, Escherichia coli O55: B5) was purchased from Sigma-Aldrich (St. Louis, MO, USA).

### 4.2. Isolation and Purification of the LCP-90-3

The detailed extraction procedure was illustrated in Figure 1. Briefly, the herb of *L. christinae* (15 kg) was firstly defatted three times with 95% ethanol for 3 h each time. Then, the residue was extracted with boiling water for 3 h (3 times). The combined water extracts were concentrated, followed by graded ethanol precipitation at final concentrations of 75% and 90%, and further deproteinated by treatment of 10% trichloroacetic acid. After neutralization, dialysis, and lyophilization, the crude fraction LCP-90 (55.93 g) was obtained. Subsequently, LCP-90 (50 g) was dissolved and loaded onto a DEAE-cellulose 52 column and then eluted by 0, 0.1, 0.2, 0.4, 0.8, and 1.6 M NaCl solution at a flow rate of 10 mL/min [16]; every 250 mL was checked at 490 nm by the phenol sulfuric acid method. The fraction eluted with 0.4 M NaCl solution was combined, concentrated, dialyzed (cut-off 3000 Da), and lyophilized to obtain a polysaccharide (LCP-90-3, 629.14 mg).

### 4.3. Structural Characterization of LCP-90-3

#### 4.3.1. Homogeneity, Relative Molecular Weight, and Chemical Analysis

The homogeneity and relative molecular weight of polysaccharide were analyzed by the high-performance gel permeation chromatography (HPGPC) method as detailed in a previous study [10]. The HPGPC system consisted of an Agilent 1200 HPLC system with a TSK GMPWXL gel filtration column (7.6 mm × 300 mm, Tokyo, Japan), a refractive index detector (RID), and an evaporative light scattering detector (ELSD). The sample solution (20 μL) was prepared by dissolving 5 mg of the polysaccharide in 2 mL of 0.02 M ammonium acetate solution, which was then injected into the HPGPC system. Dextran standards with varying molecular weights (P5, P10, P20, P50, P100, P200, P400, and P800) were used as calibration standards. The injected samples (standard or analyte) were eluted with 0.02 M ammonium acetate solution at a flow rate of 0.6 mL/min and a temperature of 30 °C. The relative molecular weight of polysaccharide was determined based on the standard curve. 

The content of uronic acid was determined by the m-hydroxylbiphenyl method using glucuronic acid (GlcA) as the standard. The total protein content was estimated by Bradford’s method using bovine serum albumin as the standard. The total carbohydrate content was determined by the phenol sulfuric acid method using mixed standards based on the results of monosaccharide composition [15].

#### 4.3.2. Monosaccharide Composition Analysis

The monosaccharide composition of polysaccharide LCP-90-3 was determined using the PMP (1-phenyl-3methyl-5-pyrazolone) pre-column derivatization method combined with HPLC analysis [31]. The Agilent 1200 HPLC system, equipped with an InertSustain C18 column (5 μm, 4.0 mm × 250 mm) (Santa Clara, CA, USA) and an ultraviolet absorbance detector (wavelength: 254 nm), was used for the analyses. The PMP derivatives were eluted by a mixture of 0.1 M phosphate-buffered solution (pH = 6.9) and acetonitrile (83: 17, *v*/*v*) at a flow rate of 0.6 mL/min.

#### 4.3.3. Fourier Transform-Infrared (FT-IR) Spectrometric Analysis

The organic functional groups of the sample were detected using an FT-IR spectrophotometer (Bruker, Ettlingen, Germany). The sample was evenly dispersed in a trichloromethane solution, coated onto KBr flake, and dried under a hot lamp prior to FT-IR measurement over a wavenumber range of 4000 to 400 cm^−1^.

#### 4.3.4. Uronic Acid Reduction, Methylation, and GC-MS Analysis

Given that the polysaccharide LCP-90-3 contains uronic acid, reduction was performed prior to methylation [32]. Specifically, 20 mg of the sample was dissolved in 60 mL of deionized water, followed by the addition of 2 mL of 1-(3-dimethylaminopropyl)-3-ethylcarbodiimide hydrochloride solution (EDC, 0.5 g/mL). The reaction mixture was maintained at pH 4.75 for 1 h. Subsequently, 8 mL of 2 M NaBH_4_ solution was slowly added, and the mixture was heated to 50 °C. The pH was adjusted to 7.0 using HCl and kept constant for 1 h. The reaction was quenched by adding acetic acid, and the solution was dialyzed against deionized water (MWCO: 3000 Da). To ensure complete reduction of the acidic polysaccharide, this procedure was repeated twice.

The methylation protocol was adapted from Hakomori’s method [33]. Preparation of sodium methylsulfinylmethanide (SMSM, 2 M): First, 5 g of NaH (60% *w*/*w* in oil) was washed three times with 25 mL petroleum ether. After decanting the supernatant each time, the residue was dried under a nitrogen stream to obtain oil-free NaH powder. Under nitrogen protection, 50 mL of anhydrous DMSO was added to the NaH powder, and the mixture was reacted at 60 °C in an oil bath for 6 h. Hydrogen gas generated during the reaction was carefully vented. The resulting grass-green 2 M SMSM solution was stored for subsequent use.

The reduced polysaccharide product was thoroughly dried overnight in a desiccator with P_2_O_5_ powder. Next, 6 mL of DMSO was added to the dried sample and stirred at room temperature until complete dissolution. Under nitrogen protection, 5 mL of the pre-prepared SMSM solution was slowly added, and the reaction proceeded overnight. Following this, 5 mL of CH_3_I was gradually introduced, and the mixture was subjected to ultrasonic treatment in an ice bath for 2 h, followed by 12 h of stirring at room temperature under nitrogen. The reaction mixture was dialyzed (MWCO: 1000 Da) for two days, extracted with chloroform, and centrifuged (5000× *g*, 10 min) to isolate the methylated LCP-90-3. FT-IR analysis confirmed complete methylation, as indicated by the disappearance of the O–H stretching band (3200–3700 cm^−1^).

The methylated product was hydrolyzed with 2 M trifluoroacetic acid (TFA) at 110 °C for 6 h. After hydrolysis, a 10 mg/mL NaBH_4_ solution was added, and the reaction proceeded at 40 °C for 30 min before termination with 100 μL glacial acetic acid. The resulting solution was concentrated to dryness, then derivatized by adding 2 mL pyridine and 2 mL acetic anhydride, followed by heating at 95 °C for 1 h. The mixture was re-dried, and the partially methylated alditol acetates (PMAAs) were extracted through repeated chloroform/water partitioning. The chloroform layer was filtered through a 0.22 μm membrane for GC-MS analysis. The glycosidic linkage patterns were determined by comparing PMAA fragmentation profiles with data from the literature [34].

### 4.4. Anti-Inflammatory Activity of LCP-90-3 in LPS-Induced ANA-1 in Vitro

#### 4.4.1. Cell Culture

Mouse macrophage cell line (ANA-1) was obtained from Procell Life Science & Technology (Wuhan, China). All cells were cultured in RPMI 1640 containing 10% FBS, 1% penicillin-streptomycin, and cultured at 37 °C with 5% CO_2_.

#### 4.4.2. Cell Viability

The cell viability test was conducted using the MTT (3-(4,5-dimethylthiazol-2-yl)-2,5-diphenylterazolium bromide) assay, with the dosage slightly adjusted as previously described in the literature [35]. Briefly, 100 μL of 2 × 10^5^ cells were cultured into 96-well plates for 24 h. Subsequently, 100 μL of LCP-90-3 was added to yield final concentrations of 500, 250, 125, 62.5, and 31.25 μg/mL, followed by a 24 h incubation. The supernatant was then aspirated, and 100 μL of MTT solution (MTT: medium, 1:4) was added, with incubation continuing for 1 h. After the incubation, the MTT solution was removed, and 100 μL of DMSO was added. The absorbance at 570 nm was measured using a microplate reader (Thermo Multiskan Go, Waltham, MA, USA).

#### 4.4.3. Determination of Inflammatory Cytokines 

ANA-1 cells were seeded into a 96-well plate at a density of 5 × 10^5^ cells/well and incubated for 24 h. The cells were treated with LCP-90-3 (62.5, 125, 250, and 500 μg/mL) for 24 h and were simultaneously stimulated with LPS (10 μg/mL). DEX (60 μg/mL) was used as a positive control [36]. The supernatant was collected and tested for nitric oxide (NO) and other inflammatory cytokines (TNF-α and IL-6) levels using enzyme-linked immunosorbent assay (ELISA) kits according to the manufacturer’s instruction (Boatman, Shanghai, China).

### 4.5. In Vivo Experiments of LCP-90-3 on LPS-Induced Acute Liver Injury in Mice

#### 4.5.1. Animals and Ethics Statement

Male C57BL/6 mice aged 8 weeks (20–22 g) were purchased from Shanghai SLAC Lab Ani-mal Co., Ltd. (Shanghai, China). The mice were housed in a controlled environment with constant temperature and humidity, specific pathogen-free conditions, and free access to food and water. All animal experiment protocols were approved by the Animal Ethical Committee of Ningxia Medical University (2024-Z064) and were conducted in accordance with the National Research Council’s Guide for the Care and Use of Laboratory Animals.

#### 4.5.2. Liver Injury Experiments of LCP-90-3

The mice were randomly divided into six groups (*n* = 6 for each group), including control, LPS, LCP-90-3-L (20 mg/kg), LCP-90-3-M (40 mg/kg), LCP-90-3-H (80 mg/kg), and silymarin (80 mg/kg). LPS (10 mg/kg) was intraperitoneally injected to induce acute liver injury in all groups except the control group, in which mice were injected with an equal volume of physiological saline [37]. The LCP-90-3 and silymarin groups were administered intragastrically once a day for 4 days before LPS injection, while the control and LPS groups received an equal volume of physiological saline. The mice’s blood and livers were collected at 24 h after LPS administration. Blood was centrifuged at 3000 rpm for 10 min to separate serum for further analysis [38]. Liver tissues were harvested and stored for subsequent testing.

#### 4.5.3. Determination of Inflammatory Cytokines and Hepatic Enzyme in Serum

The inflammatory factors (NO, TNF-α, and IL-6) in serum were detected using ELISA kits according to the manufacturer’s instructions (Boatman, Shanghai, China). The activities of alanine aminotransferase (ALT) and aspartate aminotransferase (AST) in serum were detected following the kit instructions (Jiancheng Bioengineering Institute, Nanjing, China).

#### 4.5.4. Antioxidant Activity Assay *In Vivo*

We prepared 10% left liver tissue homogenate with physiological saline solution. The levels of malondialdehyde (MDA), the activities of catalase (CAT), and superoxide dismutase (SOD) were detected according to the kit instructions (Jiancheng Bioengineering Institute, Nanjing, China).

#### 4.5.5. Histologic Examination

The formalin-fixed livers were dehydrated and embedded in paraffin (*n* = 4 for each group). After cutting into 5 μm sections, tissues were stained with hematoxylin and eosin (H&E) and examined by an Olympus VS200 slide scanner (Tokyo, Japan).

#### 4.5.6. Statistical Analysis

All data were shown as mean ± SD and analyzed by one-way ANOVA followed by Dunnett’s multiple comparisons test. GraphPad Prism 8.0 was used for all statistical analysis. A *p* value < 0.05 was considered to be statistically significant.

## 5. Conclusions

An acidic heteropolysaccharide, LCP-90-3, with anti-inflammatory activity was successfully isolated and purified from *Lysimachia christinae*. Linkage analysis further indicated that LCP-90-3 was mainly composed of 1,4-Glc*p*A, 1,4,6-Glc*p*A, 1,2-Ara*f*, T-Man*p*, 1,3-Ara*f*, and 1,4-Man*p* residues. LCP-90-3 significantly alleviated LPS-induced acute liver injury in mice. The preliminary mechanism was associated with anti-inflammatory and antioxidant effects. These findings offer valuable insights into the development of liver injury-related drugs based on polysaccharides.

## Figures and Tables

**Figure 1:**
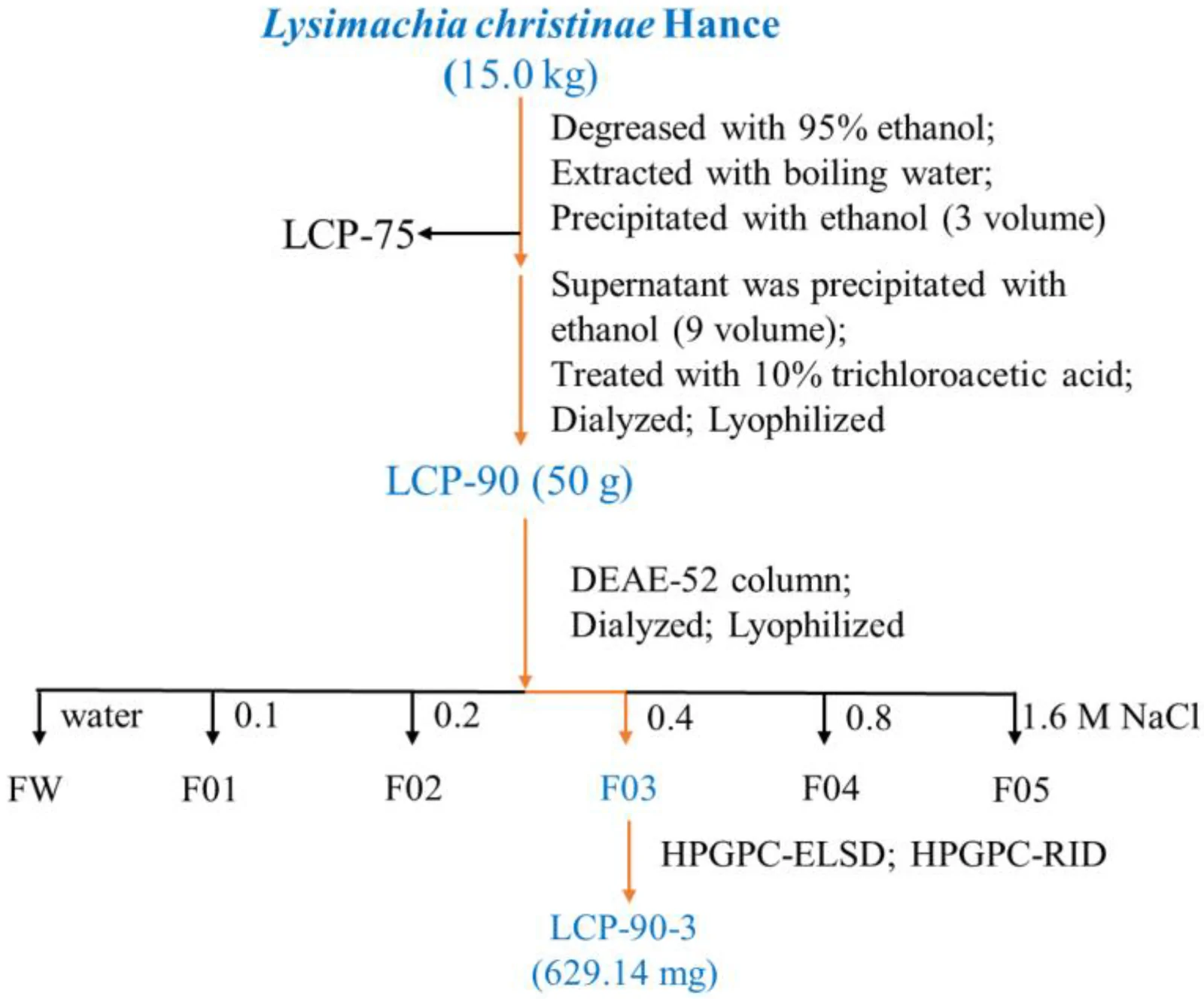
A scheme of the extraction and isolation of the polysaccharide LCP-90-3 from *Lysimachia christinae*.

**Figure 2:**
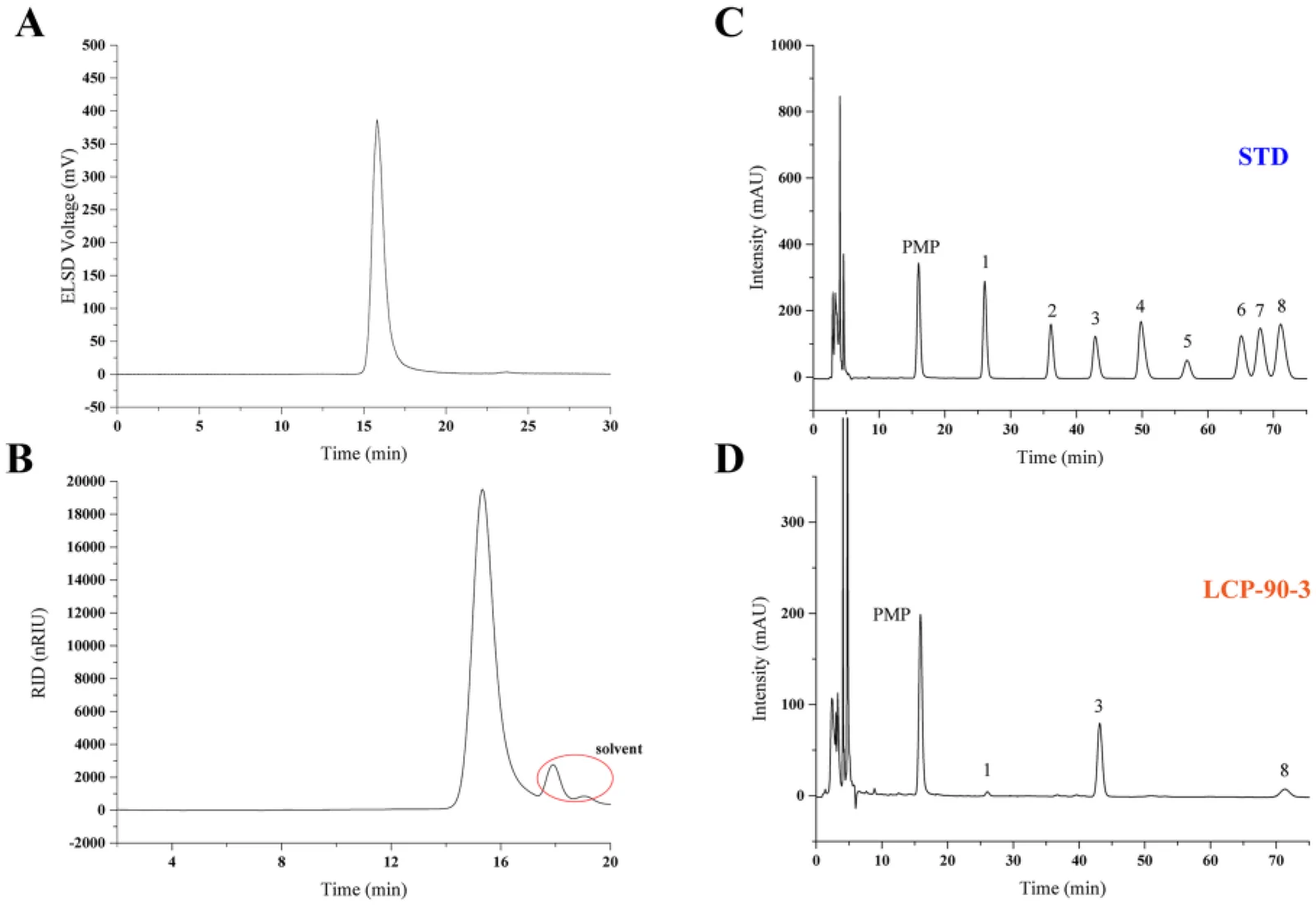
(**A**) The HPGPC-ELSD chromatogram of LCP-90-3. (**B**) The HPGPC-RID chromatogram of LCP-90-3. (**C**) HPLC spectra of mixed monosaccharide standard (1. mannose; 2. rhamnose; 3. glucuronic acid; 4. galacturonic acid; 5. glucose; 6. galactose; 7. xylose; 8. arabinose). (**D**) The HPLC spectrum of monosaccharide composition of LCP-90-3.

**Figure 3:**
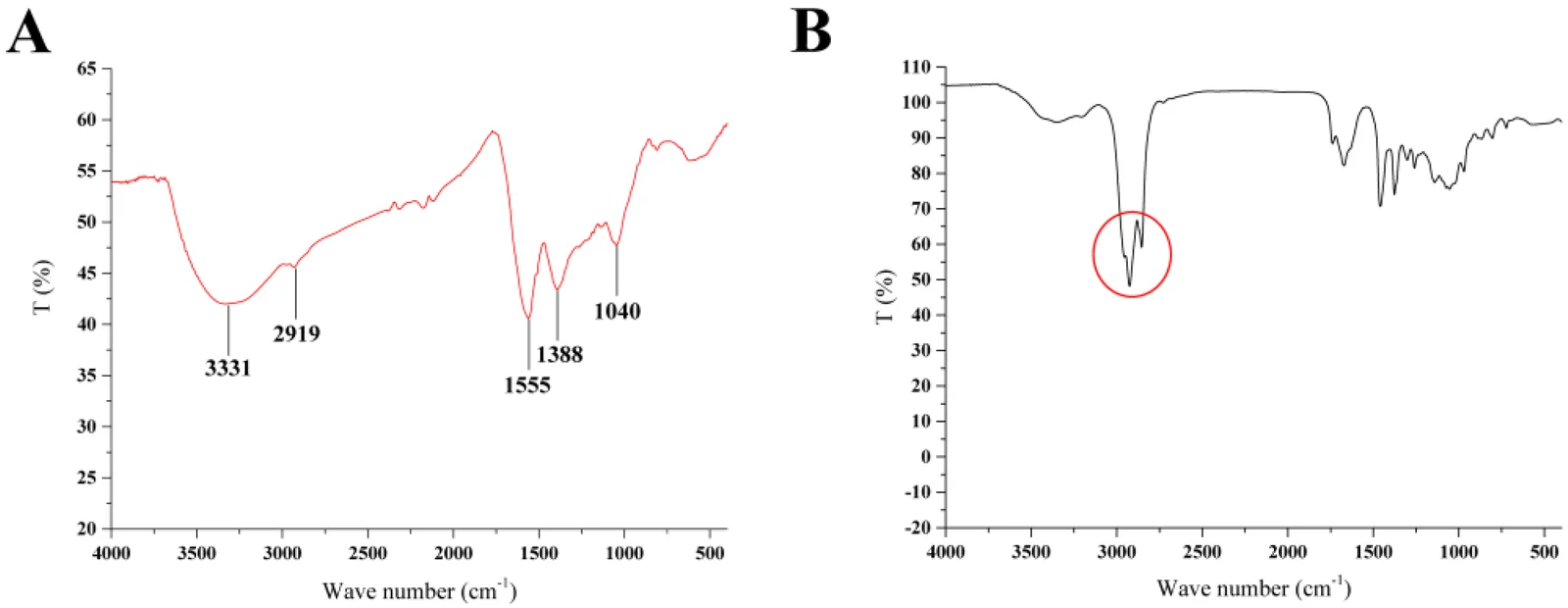
(**A**) The FT-IR spectrum of LCP-90-3. (**B**) The FT-IR spectrum of methylated products of LCP-90-3.

**Figure 4:**
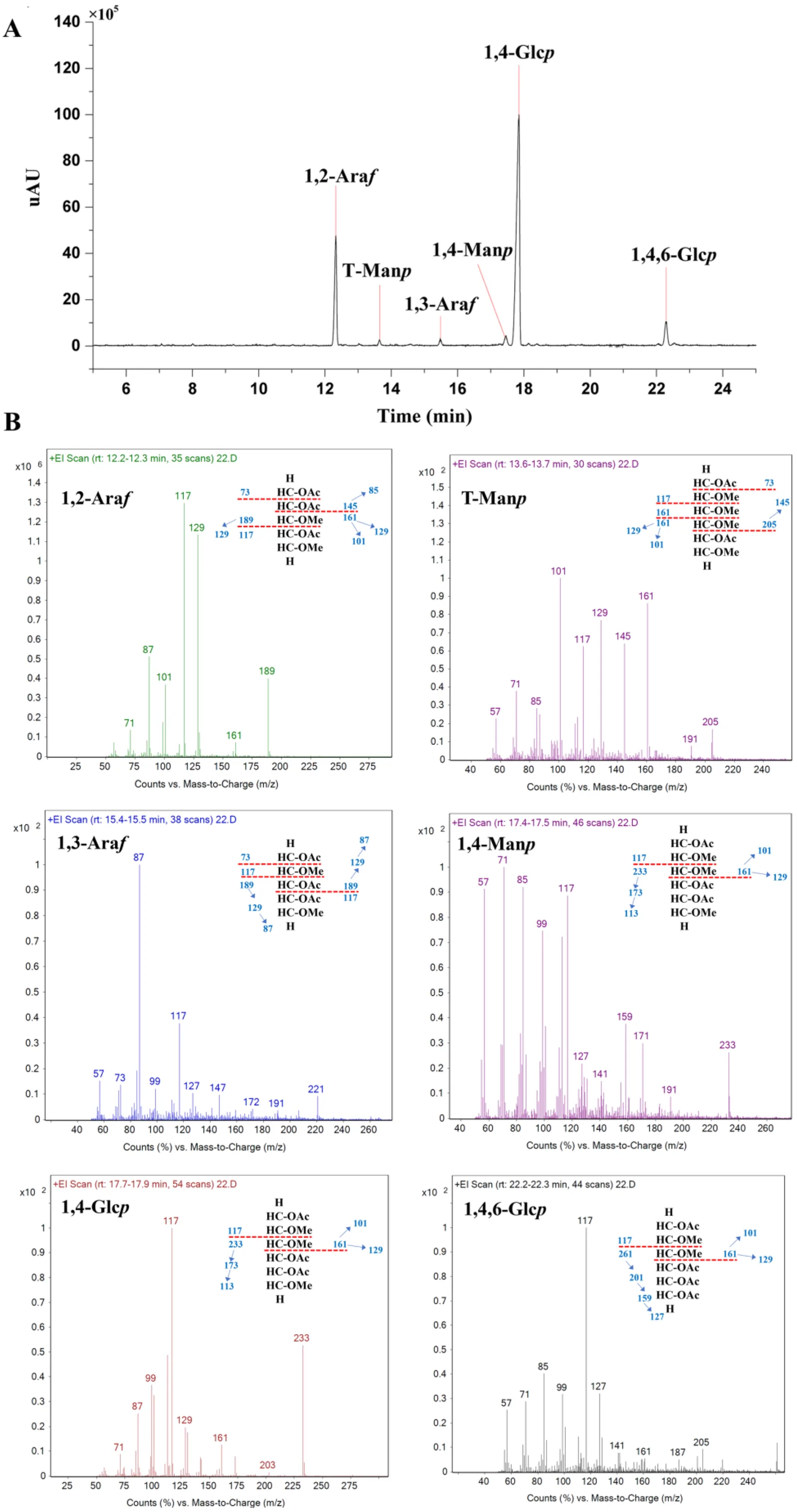
The total ion chromatography (**A**) and mass spectra (**B**) of partially methylated anhydro alditol acetates (PMAAs) of carboxyl-reduced LCP-90-3 were determined by GC–MS analysis.

**Figure 5:**
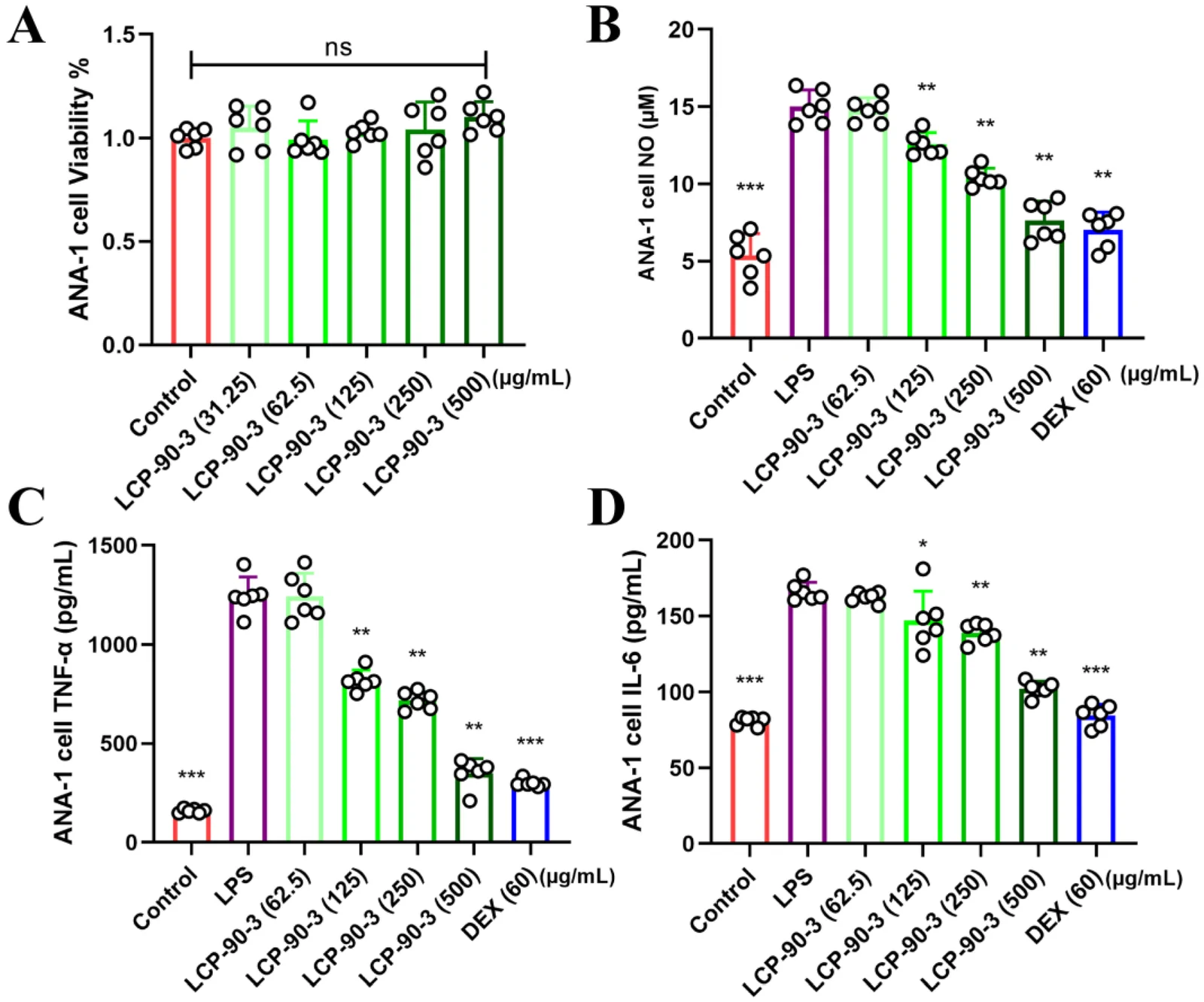
Anti-inflammatory activity of LCP-90-3 in LPS-stimulated ANA-1 *in vitro*. (**A**) Cell viability assay. (**B**–**D**) Levels of inflammatory mediators (NO, TNF-α, and IL-6) (*n* = 6). * *p* < 0.05, ** *p* < 0.01, *** *p* < 0.001 compared with LPS group.

**Figure 6:**
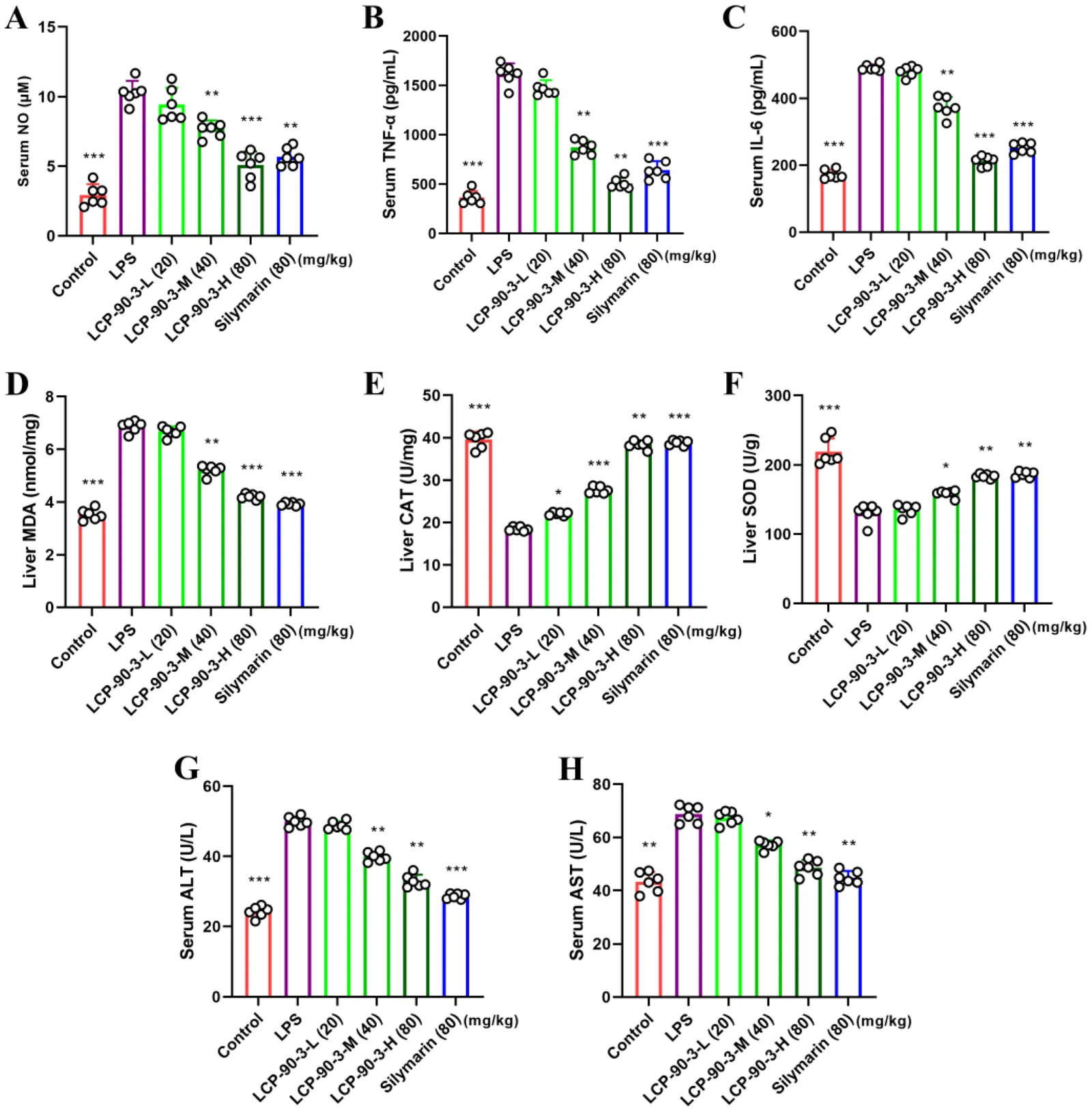
The hepatoprotective activity of LCP-90-3 in LPS-induced acute liver injury in mice. (**A**–**C**) The levels of inflammatory mediators (NO, TNF-α, and IL-6) in serum. (**D**–**F**) The MDA level and the activities of CAT and SOD in liver. (**G**,**H**) The activities of ALT and AST in serum. * *p* < 0.05, ** *p* < 0.01, *** *p* < 0.001 compared with LPS group.

**Figure 7:**
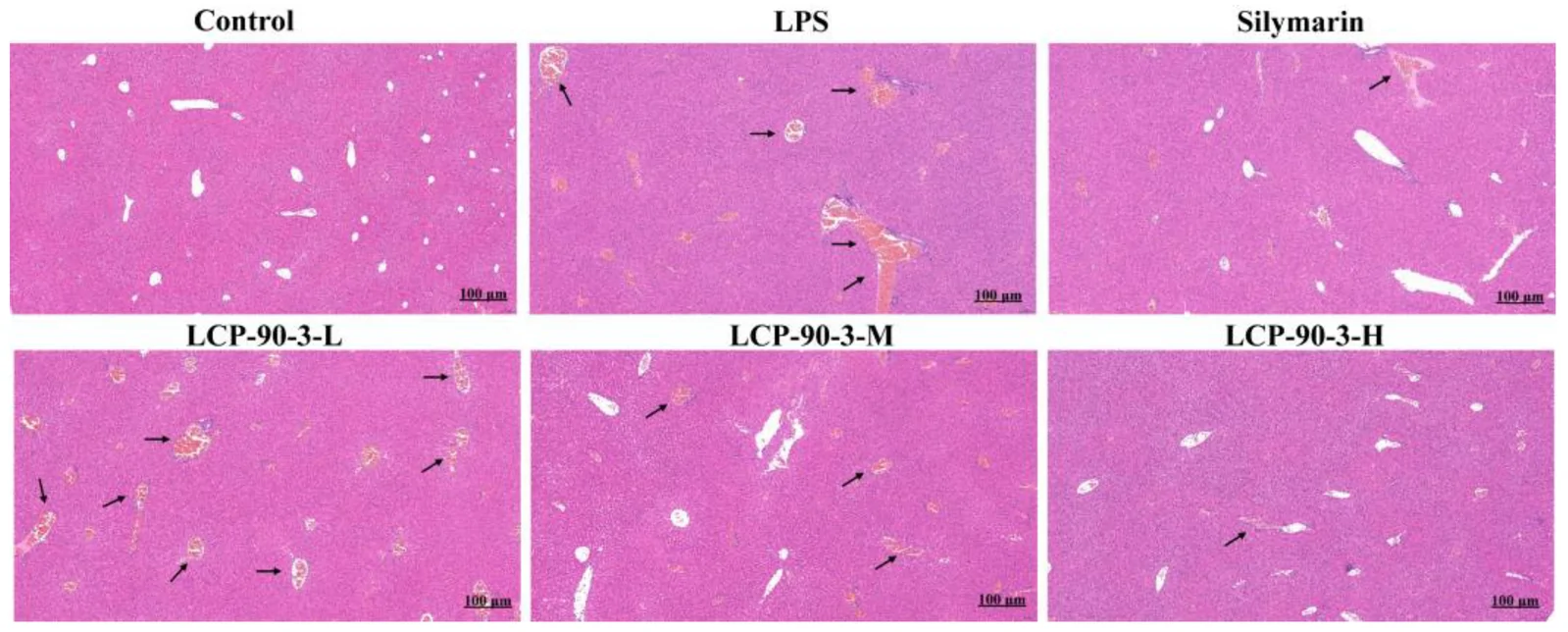
Histopathological changes in liver were analyzed using H&E staining (Bar = 100 μm). Black arrows indicate inflammatory infiltration.

**Table 1:** The GC-MS analysis of the methylated carboxyl-reduced products of LCP-90-3.

PMAAs	Linkages	Molar Ratio	Major Mass Fragments (*m*/*z*)
3,5-Me_2_-Ara*f*	1,2-Ara*f*	21.4	73, 85, 101, 117, 129, 145, 161, 189
2,3,4,6-Me_4_-Man*p*	T-Man*p*	0.9	57, 71, 101, 117, 129, 145, 161, 205
2,5-Me_2_-Ara*f*	1,3-Ara*f*	1.1	57, 73, 87, 117, 129, 189
2-Me-Ara*f*	1,4-Man*p*	2.2	101, 113, 117, 129, 161, 173, 233
2,3,6-Me_3_-Glc*p*	1,4-Glc*p*A	68.7	101, 113, 117, 129, 161, 173, 233
2,3-Me_2_-Glc*p*	1,4,6-Glc*p*A	5.7	85, 101, 117, 129, 159, 161, 201, 261

## Data Availability

Data will be provided upon request.

## Supplementary Materials

The following supporting information can be downloaded at https://www.mdpi.com/article/10.3390/molecules31193432/s1, Figure S1: The elution profile of LCP-90 on a DEAE-52 column. Figure S2: (A) Dextran standards with known weight-average molecular weights (*Mw*). (B) Calibration curve for *Mw* determination. (C) Dextran standards with known number-average molecular weights (*Mn*). (D) Calibration curve for *Mn* determination.

## Abbreviations

The following abbreviations are used in this manuscript:

ANA-1	Mouse macrophage cell line
ALT	Alanine aminotransferase
AST	Aspartate aminotransferase
CAT	Catalase
DEX	Dexamethasone sodium phosphate
ELSD	Evaporative light scattering detector
FBS	Fetal bovine serum
GlcA	Glucuronic acid
HPGPC	High-performance gel permeation chromatography
LCP-90-3	A homogenous polysaccharide isolated from *Lysimachia christinae*
IL-6	Interleukin-6
LPS	Lipopolysaccharide
MDA	Malondialdehyde
NO	Nitric oxide
RID	Refractive index detector
SMSM	Sodium methylsulfinylmethanide
SOD	Superoxide dismutase
TCM	Traditional Chinese Medicine
TNF-α	Tumor necrosis factor-α
