# Supplementary material for: Preliminary Structural Characterization of an Anti-Inflammatory Polysaccharide from Lysimachia christinae and Its Preventive Effect on Lipopolysaccharide-Induced Acute Liver Injury in Mice
Source: Molecules. 2026 Sep 27;31(19):3432. doi: 10.3390/molecules31193432 (PMC13666425; doi:10.3390/molecules31193432)
Supplement: Supplementary file 1 [file molecules-31-03432-s001.zip › molecules-4538644-supplementary.pdf]

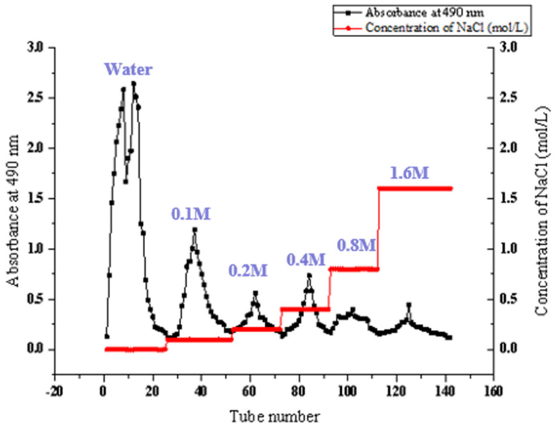


**Figure S1.** The elution profile of LCP-90 on a DEAE-52 column

A

| Standards | *Mw* | Time (min) | Log *Mw* |
|---|---|---|---|
| P800 | 642000 | 12.132 | 5.8075 |
| P400 | 337000 | 12.633 | 5.5276 |
| P200 | 194000 | 13.025 | 5.2878 |
| P100 | 107000 | 13.476 | 5.0294 |
| P50 | 47100 | 14.305 | 4.6730 |
| P20 | 21100 | 15.029 | 4.3243 |
| P10 | 9600 | 15.695 | 3.9823 |
| P50 | 6100 | 16.07 | 3.7853 |

B

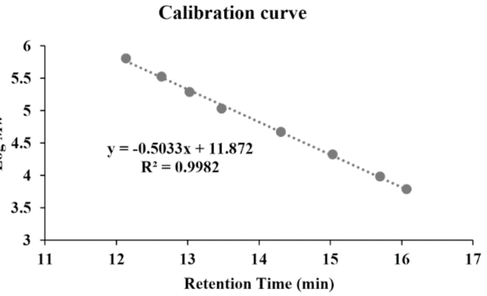


C

| Standards | *Mn* | Time (min) | Log *Mn* |
|---|---|---|---|
| P800 | 739000 | 12.132 | 5.8686 |
| P400 | 348000 | 12.633 | 5.5416 |
| P200 | 216000 | 13.025 | 5.3345 |
| P100 | 100700 | 13.476 | 5.0030 |
| P50 | 49700 | 14.305 | 4.6964 |
| P20 | 22000 | 15.029 | 4.3424 |
| P10 | 9800 | 15.695 | 3.9912 |
| P50 | 5300 | 16.07 | 3.7243 |

D

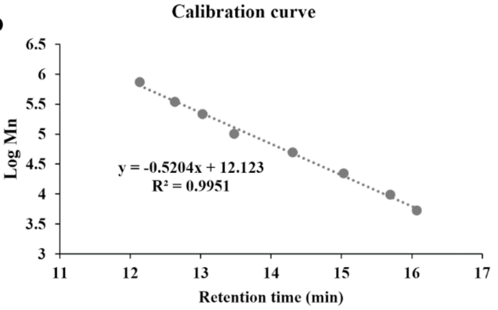


**Figure S2**. (A) Dextran standards with known weight-average molecular weights (*Mw*). (B) Calibration curve for *Mw* determination. (C) Dextran standards with known number-average molecular weights (*Mn*). (D) Calibration curve for *Mn* determination.
